# Anti-merozoite antibodies induce natural killer cell effector function and are associated with immunity against malaria

**DOI:** 10.1126/scitranslmed.abn5993

**Published:** 2023-02-08

**Authors:** Dennis O. Odera, James Tuju, Kennedy Mwai, Irene N. Nkumama, Kristin Fürle, Timothy Chege, Rinter Kimathi, Stefan Diehl, Fauzia K. Musasia, Micha Rosenkranz, Patricia Njuguna, Mainga Hamaluba, Melissa C. Kapulu, Roland Frank, on behalf of the CHMI-SIKA Study Team, In addition to the CHMI-SIKA Study Team member who is an author (Rinter Kimanthi), the following CHMI-SIKA Study Team members are collaborators who have contributed to study design, data analysis, and interpretation:, Abdirahman I. Abdi, Primus Che Chi, Zaydah de Laurent, Irene Jao, Dorcas Kamuya, Gathoni Kamuyu, Johnstone Makale, Linda Murungi, Jennifer Musyoki, Michelle Muthui, Jedidah Mwacharo, Silvia Kariuki, Daniel Mwanga, Joyce Mwongeli, Francis Ndungu, Maureen Njue, George Nyangweso, Domitila Kimani, Joyce M. Ngoi, Janet Musembi, Omar Ngoto, Edward Otieno, Michael Ooko, Jimmy Shangala, Juliana Wambua, Khadija Said Mohammed, Donwilliams Omuoyo, Moses Mosobo, Nelson Kibinge, Sam Kinyanjui, Philip Bejon, Brett Lowe, Kevin Marsh, Vicki Marsh, Yonas Abebe, Peter F. Billingsley, Betty Kim Lee Sim, Stephen L. Hoffman, Eric R. James, Thomas L. Richie, Agnes Audi, Fredrick Olewe, James Oloo, John Ongecha, Martin O. Ongas, Nelly Koskei, Peter C. Bull, Susanne H. Hodgson, Cheryl Kivisi, Mallika Imwong, Sean C. Murphy, Bernhards Ogutu, Joel Tarning, Markus Winterberg, Thomas N. Williams, Faith H. A. Osier

**Affiliations:** 1 Centre of Infectious Diseases, Heidelberg University Hospital, Heidelberg, Germany; 2 Centre for Geographic Medicine Research (Coast), Kenya Medical Research Institute–Wellcome Trust Research Programme, Kilifi, Kenya; 3 Epidemiology and Biostatistics Division, School of Public Health, University of the Witwatersrand, Johannesburg, South Africa; 4 Department of Life Sciences, Imperial College London, UK; 5 Centre for Tropical Medicine and Global Health, Nuffield Department of Medicine, University Oxford, Oxford, UK; 6 Sanaria Inc., Rockville, MD, USA; 7 Centre for Clinical Research, Kenya Medical Research Institute, Kisumu, Kenya; 8 Department of Pathology, University of Cambridge, Cambridge, UK; 9 The Jenner Institute, University of Oxford, Oxford, UK; 10 Pwani University, P. O. Box 195-80108, Kilifi, Kenya; 11 Faculty of Tropical Medicine, Department of Molecular Tropical Medicine and Genetics, Mahidol University, Bangkok, Thailand; 12 Departments of Laboratory Medicine and Microbiology, University of Washington, Seattle, WA, USA; 13 Center for Research in Therapeutic Sciences, Strathmore University, Nairobi, Kenya; 14 Mahidol-Oxford Tropical Medicine Research Unit, Mahidol University, Bangkok, Thailand; 15 Department of Medicine, Imperial College, London, UK

## Abstract

Natural killer (NK) cells are potent immune effectors that can be activated via antibody-mediated Fc receptor engagement. Using multiparameter flow cytometry, we found that NK cells degranulate and release IFN-γ upon stimulation with antibody-opsonized *Plasmodium falciparum* merozoites. Antibody-dependent NK (Ab-NK) activity was largely strain transcending and enhanced invasion inhibition into erythrocytes. Ab-NK was associated with the successful control of parasitemia after experimental malaria challenge in African adults. In an independent cohort study in children, Ab-NK increased with age, was boosted by concurrent *P. falciparum* infections, and was associated with a lower risk of clinical episodes of malaria. Nine of the 14 vaccine candidates tested induced Ab-NK, including some less well-characterized antigens: P41, P113, MSP11, RHOPH3, and *Pf*_11363200. These data highlight an important role of Ab-NK activity in immunity against malaria and provide a potential mechanism for evaluating vaccine candidates.

## Introduction

The World Health Organization recently recommended the widespread use of the RTS,S malaria vaccine in areas with moderate to high malaria transmission intensity (1). This is a major landmark because no other malaria vaccine has advanced to this stage, but RTS,S is modestly efficacious, and the protection it induces wanes rapidly (2). Vaccine development for malaria remains an urgent priority. Repeated exposure to *Plasmodium falciparum* under high transmission intensity results in long-term immunity to clinical malaria episodes in individuals living in malaria-endemic areas (3). In addition, passive transfer experiments demonstrated the importance of antibodies in mediating protection (4). However, the precise mechanisms that underpin their actions are still under investigation, and there are no universally accepted correlates of protection. Numerous studies focus on the Fab-dependent neutralization of parasites, whereby antibodies inhibit the invasion of erythrocytes by merozoites, albeit with inconsistent results (5). Antibody-dependent Fc-mediated interactions with effector cells are increasingly being recognized as important correlates of protection (6–10).

The abundance of natural killer (NK) cells in peripheral blood positions them as prime immune effectors (11). They are known to target malignant or virus-infected cells through an array of germline-encoded activation receptors (12, 13). Opsonized malaria parasites can also activate them via the low-affinity immunoglobin FcγRIIIa (CD16) (9, 14). Activation of NK cells leads to degranulation and the release of cytotoxic molecules like granzyme B as well as the secretion of proinflammatory cytokines, such as interferon-γ (IFN-γ) (15), with high plasma levels of both being associated with reduced parasitemia in vitro (16) and in cohort studies (17). Recent studies have shown that molecules in malaria-exposed plasma can target *P. falciparum*–infected erythrocytes via NK cells, leading to their destruction (9, 14). Merozoites may also be cleared similarly, because antibodies to many merozoite antigens have been associated with protection against malaria and are considered leading vaccine candidates (18, 19).

We developed an antibody-dependent NK (Ab-NK) cell assay focused on *P. falciparum* merozoites. We demonstrate that anti-merozoite antibodies induce NK cell degranulation and IFN-γ production in a largely strain-transcending fashion and enhance invasion inhibition in vitro. We study this mechanism in samples from a human malaria challenge study in adults and an independent prospective cohort study in children. We demonstrate that Ab-NK targeting merozoites is predictive of protection. We identify specific merozoite antigens that induce Ab-NK and provide a mechanistic correlate to support the prioritization of vaccine candidates.

## Results

### Ab-NK activated after coincubation with malaria-exposed plasma and merozoites

**Fig. 1:**
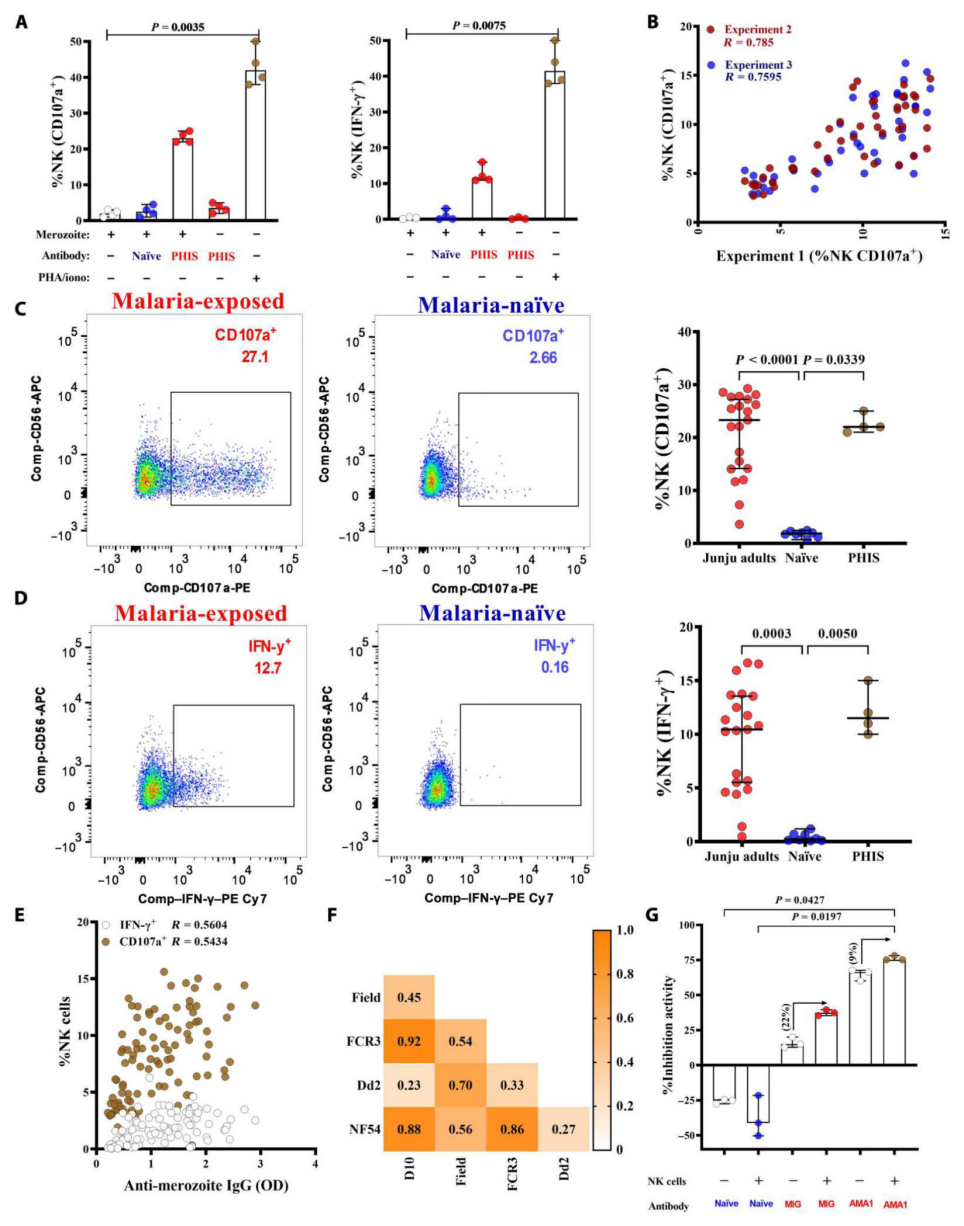
Anti-merozoite antibodies activate NK cells. (**A**) NK cell degranulation and IFN-γ production were measured by CD107a surface and intracellular staining, respectively. Merozoites were opsonized with a pool of malaria-exposed (*n* = 4) or naïve plasma (*n* = 4), with PHA/ionomycin included as a positive control. Malaria-exposed plasma samples from adults in Junju county were used to optimize the assay. Data are from four independent experiments and show the median with a 95% confidence interval (95% CI). Differences between groups are compared using the Kruskal-Wallis test. (**B**) NK cell degranulation upon stimulation with merozoites opsonized with individual plasma samples from malaria-exposed Junju adults (*n* = 30). PBMCs were collected on three separate days. The graph shows the pairwise correlation of the percentage (%) of NK cells degranulating after coincubation with merozoites opsonized with malaria-exposed plasma between independent experiments conducted on 3 separate days. The red dots show the correlation between experiments conducted on day 1 versus day 2 (experiment 2). In contrast, the blue dots show the correlation between experiments on day 1 versus day 3 (experiment 3). (**C** and **D**) Representative flow plots of NK cells incubated with merozoites in the presence of malaria-exposed or malaria-naïve plasma are shown. *P. falciparum* merozoites opsonized with plasma from individual malaria-exposed adults (red circles, *n* = 20) or malaria-naïve plasma (blue circles, *n* = 4) were coincubated with donor NK cells. An additional pool of hyperimmune sera (PHIS) was included as a positive control (brown circles, *n* = 4). This pool was prepared from Junju adults with high ELISA responses against parasite schizont lysate from the 3D7 strain of *P. falciparum*. Error bars represent 95% CI of the median values; Kruskal-Wallis test. (**E**) Pairwise correlation between the proportion of IFN-γ–secreting or IFN-γ–degranulating (CD107a^+^) NK cells activated by opsonized *P. falciparum* 3D7 merozoites and merozoite ELISA quantifying total IgG responses (OD; *n* = 142). (**F**) Spearman’s correlation heatmap between the proportion of NK cells degranulating upon activation by opsonized merozoites from five *P. falciparum* strains of different geographical origins (*n* = 20). (**G**) Viable merozoites were coincubated with uninfected erythrocytes and test immunoglobulins (1.5 ng per well) in the presence or absence of donor NK cells. Data represent the median with 95% CIs of three independent experiments.

We tested whether merozoite-specific antibodies activated NK cells from malaria-naïve donors. We measured the levels of the classical surface marker of NK cell degranulation (CD107a) and intracellular IFN-γ production. NK cells isolated from fresh peripheral blood mononuclear cells (PBMCs; immediately after blood draw) exhibited higher degranulation upon stimulation than cells obtained from frozen PBMCs (fig. S1A). Activation was dependent on the presence of both malaria-exposed plasma and merozoites (Fig. 1, A and B). Malaria-exposed, but not naïve, plasma activated NK cells in a dose-dependent fashion (fig. S1, C and D). In optimization experiments, we observed donor-to-donor variation (Spearman’s *R* = 0.55) between Ab-NK responses using the same malaria-exposed plasma (*n* = 24) and NK cells from different donors. As we had previously observed in studies on neutrophils (20, 21), the pooling of NK cells from different donors reduced this inter-assay variation (Spearman’s *R* = 0.82; fig. S2). Therefore, we pooled cells from three independent donors for each experimental run (Fig. 1B). We validated the assay further using individual samples from malaria-exposed adults from Junju county in Kenya, a region with moderate malaria transmission intensity (Fig. 1, C and D).

As expected, Ab-NK was significantly correlated with total immunoglobulin G (IgG) against merozoites (Spearman’s *R* = 0.54 and 0.56 for CD107a and IFN-γ, respectively, *P* < 0.0001; Fig. 1E). This correlation was greater for the cytophilic IgG1 and IgG3 isotypes (CD107a, *R* = 0.54 and 0.51, *P* < 0.0001) when compared with the non-cytophilic IgG2 and IgG4 (CD107a, *R* = 0.47 and 0.39, *P* < 0.0001; fig. S3, A and B).

### Ab-NK mediates responses against multiple strains of *P. falciparum*

We compared Ab-NK activity against five diverse *P. falciparum* parasite isolates using 20 individual plasma samples from malaria-exposed adults from Junju county. The pairwise coefficients compare Ab-NK activity in one parasite strain versus another and indicate the extent to which the antibodies target conserved versus polymorphic epitopes. A high coefficient suggests that antibodies from the same individuals target more conserved epitopes compared with polymorphic epitopes. Four of the five isolates were long-term laboratory-cultured strains of diverse geographical origins: NF54, FCR3, and D10 from West Africa and Dd2 from South East (SE) Asia. The fifth was isolated in Kenya (East Africa) and has recently been adapted to laboratory culture conditions and is subsequently referred to as the clinical isolate. The pairwise correlation coefficients for Ab-NK cell degranulation were significant and high between the laboratory isolates of African origin (NF54, FCR3, and D10; Spearman’s *R* = 0.86 to 0.92, *P* < 0.001) but lower when compared with the clinical isolate (Spearman’s *R* = 0.45 to 0.56, *P* = 0.010; Fig. 1F). The latter may relate to the deletion of genes after long-term parasite culture (22). The pairwise correlation coefficient between the SE Asian (Dd2) versus West African laboratory isolates (NF54, FCR3, and D10) was markedly lower (Spearman’s *R* = 0.23 to 0.33, *P* = 0.245; Fig. 1F), consistent with the distinct genomic architecture observed between parasite isolates from Asia and Africa (23, 24). Unexpectedly, a significantly higher correlation was observed between the Kenyan clinical isolate and the SE Asian laboratory strain (Spearman’s *R* = 0.70, *P* < 0.001), suggesting that these are more closely related. Similar results were observed for Ab-NK cell IFN-γ production levels (fig. S4).

### Ab-NK enhanced *P. falciparum* invasion inhibition

*P. falciparum* merozoite invasion is a complex process that occurs within a narrow time window after schizont egress. We used a modified standard invasion inhibition assay to test whether Ab-NK activity affected merozoite invasion in vitro. Donor NK cells pooled from three donors, uninfected red blood cells, and freshly isolated merozoites were cocultured in the presence or absence of purified antibodies. Malaria immunoglobulin (MIG) but not naïve antibodies inhibited parasite invasion. NK cells enhanced MIG-mediated invasion inhibition by 22%, from a median increase of 15.04 (MIG) to 37.41 (MIG + NK). However, this was not statistically significant and may have been limited by the small sample size. This assay was standardized using MIG because the concentration of MIG had been determined previously (25) and can facilitate comparisons between independent laboratories. Anti–rAMA-1 (polyclonal antibodies raised in immunized rabbits) was included as a positive control for invasion inhibition (Fig. 1G).

### Ab-NK is associated with protection after malaria challenge

We next assessed Ab-NK activity in samples collected on the day before challenge (C−1) in a controlled human malaria infection (CHMI) study in semi-immune Kenyan adults (26). The study endpoints were clinical symptoms of malaria with any evidence of malaria parasites by blood film positivity or parasitemia > 500 parasites/μl, both of which warranted immediate treatment (26). Treated volunteers were further classified into those who developed fever and those who did not (febrile versus nonfebrile). Untreated volunteers were subclassified on the basis of parasite detection by polymerase chain reaction (PCR) into PCR^−^ and PCR^+^ groups (27).

**Fig. 2:**
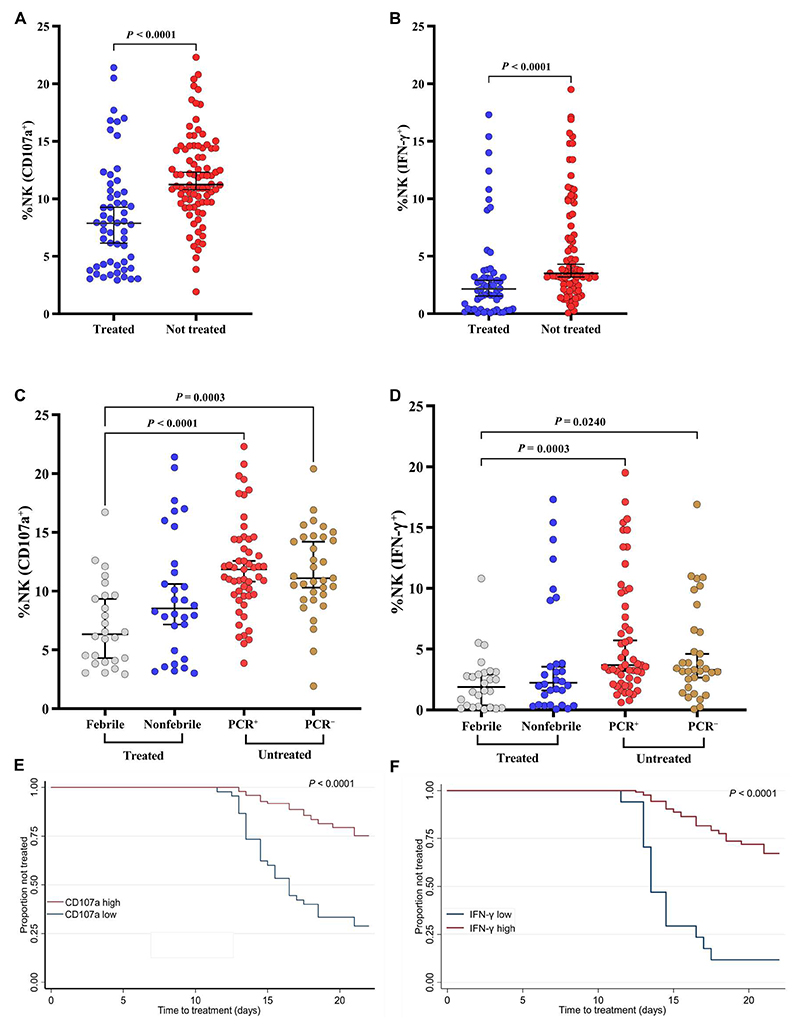
Antibody-mediated NK cell activity is associated with in vivo parasite growth. Comparison of Ab-NK cell degranulation (**A**) and IFN-γ production (**B**) in treated (*n* = 56) versus nontreated (*n* = 86) volunteers. Bars represent 95% CI of the median values; Mann-Whitney test. Subgroup analysis of Ab-NK cell degranulation (**C**) and IFN-γ production (**D**) for treated volunteers who either developed fever (febrile, *n* = 26) or did not (nonfebrile, *n* = 30) and for untreated volunteers in whom parasites were either detected by PCR (PCR^+^, *n* = 53) or remained negative (PCR^−^, *n* = 33). Bars represent 95% CI of the median values; Kruskal-Wallis with Dunn’s multiple comparisons test. Kaplan-Meier curves for the time to treatment for volunteers with a high (red) versus low (blue) Ab-NK cell degranulation (**E**) and IFN-γ production (**F**); log-rank test, *P* < 0.0001, *n* = 142.

Volunteers who were not treated (protected) had notably higher levels of Ab-NK cell degranulation and IFN-γ than those who were treated (unprotected) (Mann-Whitney *t* test, *P* < 0.00001; Fig. 2, A and B). Among the treated volunteers, there was no difference in Ab-NK levels between those who were febrile versus nonfebrile for both degranulation and IFN-γ secretion (*P* = 0.3314 and *P* = 0.9338, respectively; Fig. 2, C and D). Likewise, in the untreated (protected) subgroup, there was no difference in Ab-NK levels between those who were either PCR^+^ or PCR^−^ for both degranulation and IFN-γ secretion (*P* > 0.9999 and *P* > 0.9999, respectively; Fig. 2, C and D). However, differences in Ab-NK levels between the treated (febrile) and untreated subgroups were significant for both degranulation and IFN-γ secretion (*P* = 0.0003 and *P* = 0.0240, respectively; as shown in Fig. 2, C and D).

To test for associations with protection, we stratified Ab-NK activity into high versus low categories on the basis of threshold values derived from maximally selected rank statistics (28). The analyses were adjusted for the potential confounding effects of low levels of lumefantrine (below the minimum inhibitory concentrations) detected in the C–1 samples and the volunteers’ geographical origin (location of residence). For both degranulation and IFN-γ production, high Ab-NK activity was associated with a significantly lower risk of treatment [incidence rate ratio (IRR) of 0.41 and 0.49, respectively; *P* < 0.001; Table 1]. Furthermore, the time to detection of clinical malaria (study endpoints) after the 10th day post-challenge was longest in volunteers with high compared with those with low levels of Ab-NK activity (Fig. 2, E and F). Similarly, in a Cox regression analysis, high levels of phagocytosis were strongly associated with a longer time to detection of clinical episode hazard ratio (HR) of 0.25 and 0.22 for degranulation and IFN-γ production, respectively (*P* < 0.001; Table 1).

We further stratified the Ab-NK responses based on two clusters (top versus bottom cluster) that we observed within the treated group (bottom clusters, <5% NK cell CD107a and <0.5% NK IFN-γ; Fig. 2, A and B). Individuals in the bottom clusters had significantly lower total antibodies against the whole merozoites compared with those in the top cluster (Mann-Whitney test, *P* < 0.0001; fig. S5A). Next, we explored whether the low levels of antibodies would affect in vivo parasite growth after challenge. A subanalysis of the survival estimates within the treated group showed that the time to treatment was prolonged by 2 and 3 days, respectively, for individuals in the top cluster compared with those in the bottom cluster of Ab-NK cell CD107a and IFN-γ responses (log-rank test, CD107a: *P* = 0.0003 and IFN-γ: *P* = 0.0003; Table 1 and fig. S5, B and C).

### Not all potential merozoite vaccine candidate antigens induce Ab-NK

**Fig. 3:**
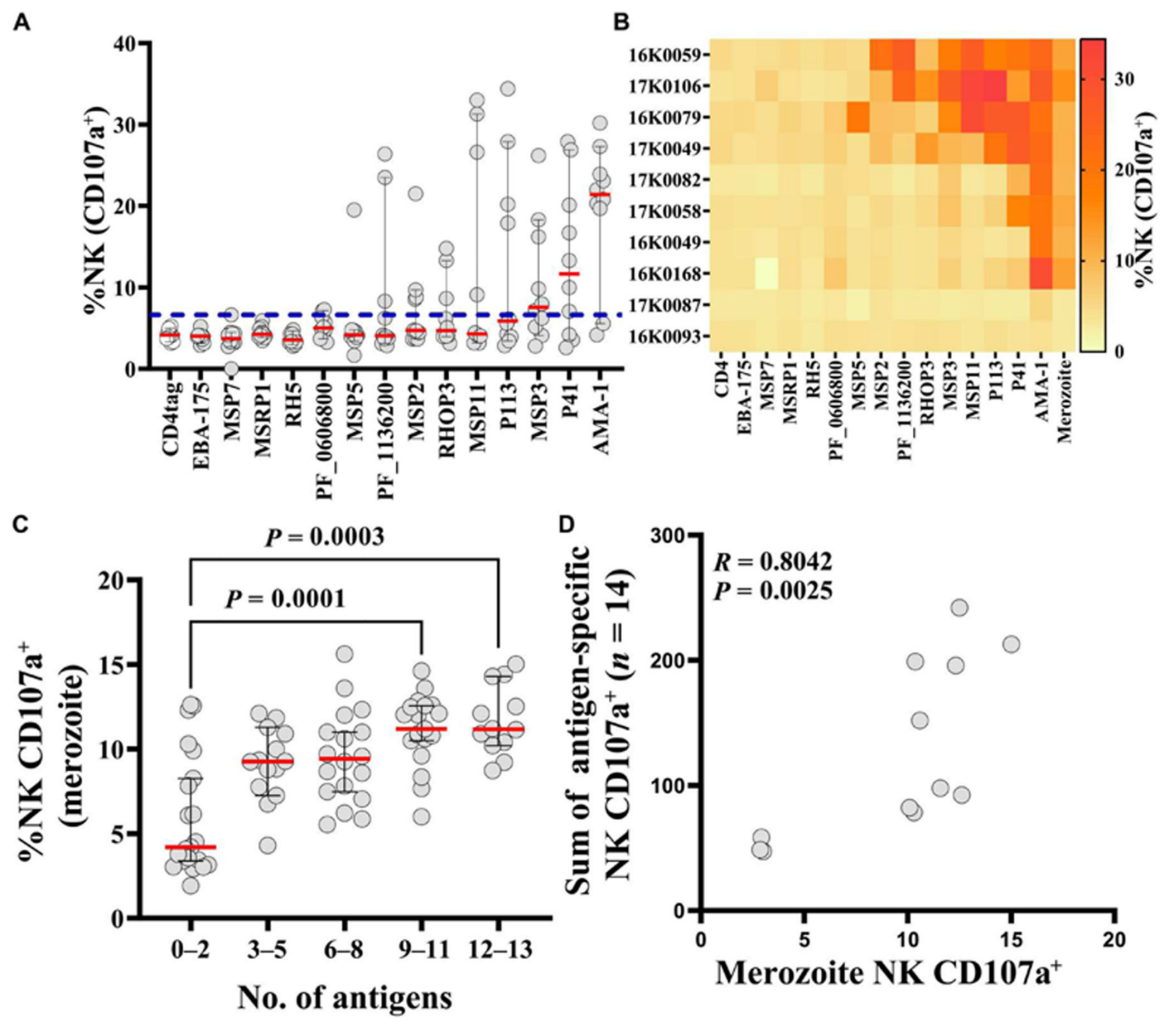
Potential targets of antibodies that mediate Ab-NK. (**A**) Antigen-specific Ab-NK cell degranulation from a subset of CHMI individuals (*n* = 10) against 14 unique recombinant *P. falciparum* merozoite antigens. The dashed line represents the cutoff value based on the mean plus 3 SDs of the tagged fragment (CD4 tag). Error bars represent 95% CI of the median (red line). (**B**) Heatmap of individual level antigen-specific Ab-NK cell degranulation from (A). Each row represents a single CHMI participant, and each column represents responses to a single recombinant merozoite surface–associated antigen. (**C**) Ab-NK cell degranulation against whole merozoite extract was associated with the breadth of antigen-specific recognition. Error bars represent 95% CI of the median (red line) *n* = 142; Kruskal-Wallis test. (**D**) The sum of antigen-specific Ab-NK cell degranulation against 14 unique recombinant merozoite antigens was correlated with NK cell degranulation against merozoites; Spearman’s correlation *R* = 0.80.

To distinguish distinct merozoite antigens that induce Ab-NK activity from those that potentially did not, we developed a plate-based Ab-NK assay using recombinant antigens. We could thus detect and quantify antigen-specific Ab-NK activity. We tested Ab-NK activity for 14 *P. falciparum* merozoite surface–associated antigens identified as potential vaccine candidates in a previous study (18). This analysis was conducted in a subset of CHMI samples with high (*n* = 8) or low (*n* = 2) concentrations of Ab-NK activity against whole merozoite extract. We found that antigen-specific Ab-NK activity varied between antigens and individuals. We identified 8 potential merozoite targets of Ab-NK activity of the 14 tested (Fig. 3A). These included well-characterized vaccine candidates like AMA-1, MSP3, and MSP2 and less-well studied antigens like P41, P113, MSP11, RHOPH3, and *Pf*_11363200 that have independently been associated with clinical protection (Fig. 3A) (18, 29, 30). Responses against other leading blood-stage vaccine candidates, such as *Pf*Rh5 and EBA-175, had negligible Ab-NK activity. Although we had preselected individuals with high Ab-NK cell degranulation against whole merozoite extract, this did not always translate into high levels of antigen-specific Ab-NK activity. This suggests that additional antigens that were not tested here contributed to the functional response against the whole merozoite (Fig. 3B). In addition, we and others previously found that the breadth of the antibody response was associated with enhanced function (19, 31, 32). In keeping with this, whole merozoite extract Ab-NK cell degranulation and the breadth of antigen-specific Ab-NK cell degranulation were strongly correlated (Fig. 3, C and D).

### Ab-NK increases with age after natural infections

**Fig. 4:**
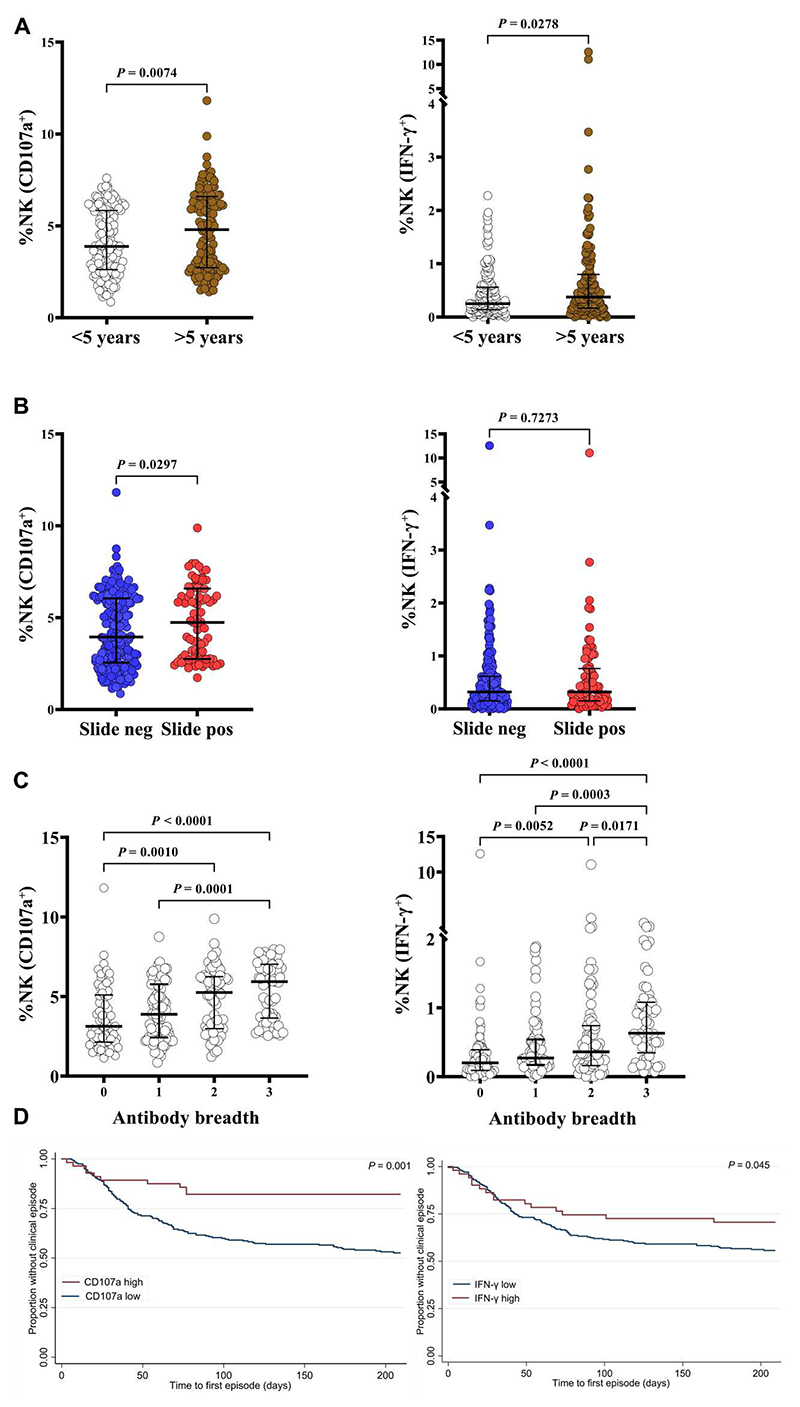
Antibody-mediated NK cell degranulation is associated with clinical protection in a prospective cohort study in Kenyan children. (**A**) Comparison of Ab-NK cell degranulation (left) and IFN-γ production (right) in Junju children over or under 5 years of age. (**B**) Ab-NK response in children who had an active *P. falciparum* infection compared with those without. Error bars represent 95% CI of the median values; Mann-Whitney test (*n* = 293). (**C**) Antibody-mediated NK cell degranulation (left) and IFN-γ production (right) were correlated with the breadth of total IgG responses against recombinant MSP2, MSP3, and/or AMA-1. Error bars represent 95% CI of the median values; Kruskal-Wallis test with Dunn’s multiple comparisons test (*n* = 293). (**D**) Ab-NK cell degranulation and IFN-γ production were associated with clinical protection against symptomatic malaria in children. Ab-NK responses were categorized as high or low (blue) on the basis of a threshold (28); log-rank test, *P* = 0.001 (*n* = 293). Each dot represents a technical replicate.

We next examined the acquisition of antibodies that mediated NK cell activity in 293 samples collected from a longitudinal cohort study of children living in Junju, Kenya (33). At the time of sampling, 67% of the children had antibodies that mediated NK cell degranulation, whereas only 10% had antibodies that induced IFN-γ production, respectively. Seropositivity was determined as Ab-NK responses higher than the mean plus 3 SDs of responses from malaria-naïve controls (Table 2). We observed lower Ab-NK activity in children compared with that in adults living in the same location (fig. S6A). Ab-NK increased with age (Fig. 4A and fig. S6B). In addition, children who were parasite slide positive at the time of sampling showed higher NK cell degranulation but not IFN-γ production than slide-negative children, suggesting a boosting effect of Ab-NK activity by active *P. falciparum* infection (Fig. 4B). Antibody responses against three malaria vaccine candidates (AMA-1, MSP2, and MSP3) had been measured in this cohort in a previous study (33). Ab-NK activity in children was modestly correlated with antibody recognition of AMA-1 (CD107a^+^: *R* = 0.35 and IFN-γ^+^: *R* = 0.31), MSP2 (CD107a^+^: *R* = 0.28 and IFN-γ^+^: *R* = 0.35), and MSP3 (CD107a^+^: *R* = 0.25 and IFN-γ: *R* = 0.24; Spearman’s *R* for all comparisons *P* < 0.0001; fig. S6C). However, despite the modest correlation, the breadth of antibody recognition against the three merozoite antigens was associated with increased NK cell activity against the whole merozoite in children, again suggesting an additive effect when multiple targets are considered (Fig. 4C).

### Ab-NK is associated with protection against clinical episodes of malaria in children

To date, no longitudinal cohort study has assessed the relationship between Ab-NK activity against merozoites and protective immunity in children living in Kenya. We stratified Ab-NK activity into high versus low categories on the basis of derived thresholds (28) and fitted them to a modified Poisson regression analysis, adjusting for age and previous *P. falciparum* exposure as confounders (6). We found that both degranulation (CD107a) and IFN-γ production were significantly associated with protection against symptomatic episodes of malaria CD107a [IRR: 0.40; 95% confidence interval (95% CI), 0.22 to 0.71; *P* = 0.002] and IFN-γ (IRR: 0.61; 95% CI, 0.39 to 0.97; *P* = 0.038; Table 3). These findings were confirmed in a Cox regression analysis in which the time to the first malaria episode was analyzed as the outcome variable (log-rank test, *P* < 0.0001; Fig. 4D).

## Discussion

Defining the mechanisms that underpin the potent efficacy of MIG observed in passive transfer experiments provides strong evidence for rational vaccine development. We report that anti-merozoite antibodies engage Fc receptors on NK cells, unleashing potent antiparasite effector activity. Ab-NK activity was associated with clinical protection in two independent studies and induced responses against multiple parasite strains, including a recently adapted clinical isolate. In addition, we identify a subset of merozoite antigens that induce Ab-NK activity and show that the breadth of antigen-specific Ab-NK activity mirrors that measured against merozoites. Our data augment the accumulating body of evidence that targeting combinations of antigens may be instrumental for malaria vaccine design (18, 19, 32, 34–38). We also provide a mechanism for the prioritization and subsequent evaluation of vaccine candidates.

Although several immune mechanisms have been proposed as correlates of protection, none have focused on Fc antibody–dependent targeting of merozoites by NK cells. The growth inhibition assay is considered the gold standard for assessing antibody function and is thought to assess a combination of invasion inhibition, growth inhibition, and possibly merozoite egress (39–42). Unfortunately, it does not reliably predict protection. Other studies have investigated the role of complement (7), monocytes (6), and neutrophils (8), marking the increasing importance of Fc-mediated function (10), with promising results that need further validation in additional studies. Our challenge study allowed us to overcome many of the limitations of cohort studies that often lead to conflicting results when any functional correlate of protection is considered. The timing and intensity of our experimental parasite challenge were accurately defined, and close monitoring for clinical symptoms and parasite multiplication rates was feasible because volunteers were hosted at a single location for the duration of the study. We included adults from a single East African country to minimize genetic variability. We preselected individuals with a varied range of previous malaria exposure to allow us to explore its impact on the clinical outcome. Under these stringent conditions, we observed a strong correlation between Ab-NK activity and protection. We found similar results in an independent study involving only children that used an entirely different design in which malaria infections occurred naturally under real-life conditions.

NK cells have been extensively studied for their role in the early defense against viral infections and cancers. In addition, the modulation of their function underpins an array of contemporary immunotherapeutic agents (43, 44). Our study capitalizes on the ability of the FcγRIIIa (CD16) to induce NK cells in response to antibody-coated targets without engaging other activating or inhibitory receptors (45). The potential impact of this mechanism on vaccines against infectious diseases is relatively understudied. Recently, a human monoclonal antibody (mAb) against a conserved region of the hemagglutinin (HA) protein was shown not only to potently neutralize a broad range of influenza viruses but also to mediate antibody-dependent cellular cytotoxicity (ADCC) (46). In HIV, evidence that ADCC-mediating antibodies complement neutralizing functions is accumulating (47, 48).

In malaria, NK cells were identified as part of the early innate immune response by producing IFN-γ and soluble granzymes (15). We used pools of malaria-naïve NK cells to interrogate their interaction with anti-merozoite antibodies. Our experimental design thus limits our interpretation to variation between the levels of anti-merozoite antibodies between study participants. NK cells are diverse, and their function and phenotypes have been shown to vary in an array of infections and ages (49). Recent studies suggest that NK cells undergo phenotypic differentiation after repeated malaria exposure. For example, they can acquire a memory-like “adaptive” phenotype after repeated exposure to malaria (14, 50), or the frequency of the CD56 negative CD16 positive phenotype can increase with repeated exposure (51, 52). Both these phenotypes mediate enhanced ADCC, suggesting that we may have observed even higher Ab-NK activity if we had used NK cells from malaria-exposed adults. Further studies to test this hypothesis and to examine Ab-NK activity using autologous NK cells are planned.

As shown for malaria antigens exposed on the surface of infected red cells (9, 50, 53), antibodies against merozoites induce Fc receptor–dependent NK activation. In our studies, antibodies inducing Ab-NK activity increased with age, with children inducing lower Ab-NK activity than adults. Ab-NK cell CD107a but not IFN-γ was boosted in the presence of an active malaria infection defined by a positive blood smear. This may reflect technical limitations in the detection of Ab-NK in the lower range of the assay. It may also reflect the limitations of the cohort study design in which the length of the infection and hence the opportunity to boost responses cannot be ascertained. However, one might expect this to apply to both Ab-NK cell CD107a and Ab-NK IFN-γ. These differences require further investigation.

Our findings are broadly in agreement with the systems serology of RTS,S–vaccinated and CHMI participants. Vaccination with RTS,S induced multifunctional antibodies that mediated phagocytosis and could engage Fc gamma receptors, and this interaction predicted protection in two human challenge studies (10, 54). However, engagement implies but does not actually demonstrate effector function. Our study goes further to show engagement and demonstrate function that was associated with reduced parasite growth in vivo in adults and protection against symptomatic episodes of malaria in children. Moreover, a systematic transcriptomic analysis of adults vaccinated by irradiated sporozoites highlighted temporal differences in genes associated with NK cells that correlated with clinical protection, further supporting their important role in parasite immunity (55).

Detecting functional activity against a diverse panel of parasite strains is noteworthy and a vitally important consideration when new assays and vaccine candidates are assessed (5, 38). Previous studies investigating antibody-dependent NK effector function in *P. falciparum* focused on a single laboratory strain (9, 14, 50, 53). We found that Ab-NK responses against merozoites from diverse *P. falciparum* strains originating in Africa were strongly correlated. Although this suggests that some of these antibodies target conserved epitopes, further confirmation at the antigen and epitope level is needed. The weaker correlations between the African laboratory versus clinical isolate indicate that some antibodies do target polymorphic epitopes. In influenza, ADCC-mediating antibodies target conserved epitopes in the HA protein (56), raising the possibility of broadly protective universal influenza vaccines.

We developed a high-throughput plate-based antigen-specific Ab-NK cell assay (57) to better identify specific merozoite antigens targeted by Ab-NK. We tested a panel of merozoite antigens that were available to us from previous studies in a subset of samples from adults (58). We observed high interindividual variation and unique Ab-NK antigen-specific reactivity profiles in each sample. This was not unexpected given the heterogeneity of antibody responses against malaria antigens that we, and others, have previously reported (18, 59). Leading vaccine antigens known to induce invasion-inhibitory antibodies, such as apical membrane antigen 1 (AMA-1) (60), or Fc-dependent activity in phagocytosis, such as MSP3 (6), were also targeted by Ab-NK in some, but not all, individuals. Less well-studied vaccine candidates such as *Pf*113 and P41 also induced Ab-NK in a proportion of individuals. Some individuals elicited high levels of Ab-NK against the whole merozoite but not against a broad range of tested antigens. Further dissection of antigen and epitope specificity is warranted and may be instructive for vaccine design.

Previous studies have demonstrated that the breadth of the antibody response against selected parasite antigens is an important predictor of protection against malaria (18, 19, 32, 34–38, 61). The breadth of antibody reactivity against the 14 *P. falciparum* antigens tested in the current study was associated with higher Ab-NK activity against the whole merozoite. This mirrors observations from other functional studies in which the breadth of the antigen-specific functional Fc antibody–dependent response was correlated with protection (18, 19). These data suggest that an effective malaria vaccine modeled on naturally acquired immunity may not only need to incorporate multiple antigens but also induce antibodies that trigger diverse effector functional activity. The influenza example is a case in point; a multifunctional human mAb was identified that simultaneously neutralizes the virus and induces ADCC (46).

We identify antibody-mediated NK cell activity targeting merozoites as a strong predictor of naturally acquired immunity against *P. falciparum* malaria. Our antigen-specific assay facilitates throughput and will galvanize additional studies focusing on specific vaccine candidates. Further dissection of Ab-NK–inducing antibodies at the epitope level may reveal important signatures for universal vaccine design.

## Materials and Methods

### Study design

In this study, we coupled the knowledge from passive transfer studies that demonstrate a key role for antibodies in conferring protection against malaria with the fact that NK cells are abundant in the peripheral blood of humans and have been shown to be important immune effectors. We leveraged the growing body of evidence that antibody-dependent Fc-mediated interactions with immune effectors are important correlates of protection and tested this hypothesis in the context of NK cells. We first established and optimized an Ab-NK cell assay that targeted *P. falciparum* merozoites and tested whether it enhanced the antibody-mediated inhibition of erythrocyte invasion. We then used plasma samples from an interventional clinical trial (CHMI) and an observational prospective cohort study (Junju cohort) to test whether Ab-NK was associated with protection against malaria. Last, we developed a plate-based assay that enabled the detection of antigen-specific Ab-NK activity using distinct recombinant merozoite antigens. The results of the fully optimized Ab-NK cell assays were reproduced in four independent experiments that included biological replicates. Human samples from the clinical cohorts were tested in single experiments, in duplicate, with appropriate positive and negative controls. Sample processing and data collection in the laboratory were blinded in relation to the clinical outcome of participants. Data were unblinded for analysis.

### CHMI ethical statement

The CHMI study was conducted at the KEMRI Wellcome Trust Research Programme in Kilifi, Kenya, with ethical approval from the KEMRI Scientific and Ethics Review Unit (KEMRI//SERU/CGMR-C/029/3190) and the University of Oxford Tropical Research Ethics Committee (OxTREC 2-16). All participants gave written informed consent. The study was registered on ClinicalTrials.gov (NCT02739763) and conducted on the basis of good clinical practice and under the principles of the Declaration of Helsinki.

### Junju ethical statement

Ethical approval for the Junju study was provided by the Kenyan National and Scientific Ethics Review Committee protocol number 3149.

### CHMI study

The CHMI study was open, unblinded, and nonrandomized. The detailed protocol and experimental approach have been published previously (26). Briefly, a dose of 3200 infectious, cryopreserved *P. falciparum* NF54 sporozoites (Sanaria *Pf*SPZ) was administered intravenously to 161 consenting Kenyan adults (18 to 45 years) with varying degrees of prior exposure to malaria [based on enzyme-linked immunosorbent assay (ELISA) responses to crude *P. falciparum* 3D7 schizont lysate].

### Study monitoring and exclusion criteria

Testing for parasitemia was conducted twice daily from day 7 to 14 and once from day 15 to 21 after challenge by quantitative PCR. Participants were treated either when they had more than 500 *Pf* /μl, exhibited clinical symptoms, and had any parasitemia or at the end of the active follow-up period on day 21. Seven of the 161 volunteers were found to have non-*Pf*NF54 stain (strain used to challenge) based on MSP2 genotyping and were excluded from further analysis (*n* = 7). An additional 12 volunteers were excluded from the analysis because when we measured their plasma levels for lumefantrine retrospectively 7 days after challenge, their plasma anti-malarial drug concentrations levels were higher than the minimum inhibitory concentration. We, therefore, excluded the samples of these individuals (*n* = 12) from further analysis (27). Thus, 142 of the 161 challenged volunteers were considered for further analysis. Among the remaining volunteers, a proportion (*n* = 64) had low lumefantrine levels, i.e., below the minimum inhibitory plasma concentrations. Data from these individuals (*n* = 64) were included in the downstream analysis. To minimize any potential confounding when assessing the association with protection, we included low levels of lumefantrine (*n* = 64) as a confounder in the multivariate regression analysis. We analyzed 142 plasma samples collected 1 day before the challenge (C–1) to test whether Ab-NK activity was associated with a lower risk of clinical malaria or parasitemia > 500 *Pf*/μl.

### Junju cohort: Observational prospective cohort study

The Junju cohort is an ongoing longitudinal study that was initially established in 2005 as part of a malaria vaccine trial (62), and participants have been followed up since then. Briefly, we analyzed 304 plasma samples collected at the beginning of the malaria transmission season in May 2008 from children aged between 0 and 12 years and residents in Junju village, Kilifi County. The *P. falciparum* parasite rate standardized to the age group 2 to 10 years (*Pf*PR_2–10_) by blood smears was 29% at the time of sampling. Active follow-up involved weekly visits to participants’ homes, where a questionnaire for clinical symptoms was administered and the temperature was recorded. In addition, study personnel were residents in the village and were contacted by caregivers at any time if the children were unwell (passive case detection). A symptomatic episode of malaria was defined either as a temperature of >37.5°C plus any parasitemia by blood smear (for children under 1 year) or a temperature of >37.5°C plus a parasite density of >2500 *Pf*/μl determined from a thick blood smear (for children older than 1 year) (63). In this study, we analyzed data that spanned a malaria transmission season of about 6 months in duration in 2008.

Independent samples collected from adults in Junju (*n* = 40) were used to establish and optimize the Ab-NK cell assay. A pool of hyperimmune sera (PHIS) collected from malaria-exposed adults from Kilifi, Kenya, and an MIG (25) was used for validation experiments and as a positive control.

### Cultivation, purification, and quantification of viable *P. falciparum* merozoites

*P. falciparum* laboratory-adapted strains of West African (NF54, FCR3, and D10) and SE Asian origin (Dd2), as well as a recently adapted clinical isolate from Kilifi, Kenya (P0000072), were cultured in RPMI 1640 medium supplemented with 0.5% AlbuMAX, gentamycin (25 μg/ml), hypoxanthine (50 μg/ml), 2 mM L-glutamine, and 25 mM Hepes buffer. Cultures were maintained at 2% parasitemia with O-positive erythrocytes obtained from malarianaïve donors (less than 2 weeks old). Free viable merozoites were isolated as previously described (64). Briefly, *P. falciparum* cultures were allowed to attain 10 to 15% parasitemia and synchronized at the ring stage using 5% sorbitol. After 24 hours, late-stage pigmented trophozoites were harvested by magnetic-activated cell sorting (MACS) column purification as per the manufacturer’s instructions (Miltenyi Biotec), attaining trophozoite purifications of 80 to 95%. The enriched trophozoites were put back in culture and allowed to develop until they began to undergo schizogony (segmented nuclei), whereby 1 mM protease inhibitor (E64; Sigma-Aldrich) was added to allow maturation into late schizonts but inhibit rupture. Mature schizonts were collected and passed through a 1.2-μm microfilter previously blocked in 1% casein in phosphate-buffered saline (PBS) for 10 min to release free viable merozoites. These were either immediately used in the modified invasion inhibition assay (IIA)–ADCC assay or washed twice in PBS, quantified, and stored at −80°C until needed. The relative concentration of the stored merozoites was determined using CountBright absolute counting beads per the manufacturer’s instructions. Briefly, 50 μl of counting beads with a known concentration were mixed with a known volume of merozoites stained with 100 μl of 1× SYBR dye for 15 min at 24°C and acquired on a BD FACSCalibur II flow cytometer. Merozoites were resuspended in 1× PBS at a working concentration of 5 × 10^7^ merozoites/ml and stored at −80°C.

### Recombinant expression of *P. falciparum* merozoite antigens

The recombinant merozoite antigens were expressed in-house as previously described (58). Briefly, Expi293F cells (Invitrogen) were cultured to a density of 2.0 × 10^6^ cells/ml and transfected with expression vectors using the Expifectamine 293 transfection reagent (Invitrogen). Cells were then incubated at 37°C with 8% CO_2_ in an orbital shaker at 125 rpm. Culture supernatants were harvested 6 days after transfection, and proteins were purified using nitriloacetic acid bound with nickel (Ni-NTA) purification columns (Invitrogen).

### Enzyme-linked immunosorbent assay

A standardized ELISA protocol was performed as published (6, 32, 33, 65). Briefly, a predetermined concentration of recombinant *P. falciparum* recombinant antigens (30 ng per well) or free whole merozoites (5 × 10^5^ merozoites per well) were coated overnight at 4°C. The plates were washed with PBS, followed by blocking with 1% casein in PBS (Invitrogen) for 2 hours at 37°C. After four washes, 50 μl of test plasma sample diluted 1:20 in PBS were added to the plates in duplicate and incubated for 1 hour at 37°C, followed by four additional washes. The plates were incubated for 1 hour at 37°C with 100 μl of secondary anti-human IgG, IgG1, IgG2, IgG3, or IgG4 (Invitrogen) antibodies used to determine total IgG or IgG isotype–specific antibody responses, respectively. Plates were washed, and 100 μl of O-phenylenediamine dihydrochloride (OPD) substrate (Invitrogen) in PBS were added and incubated for 20 min for color development. The reaction was stopped with 1 M hydrochloric acid solution (Sigma-Aldrich), and optical density (OD) was quantified at 492 nM. Serum samples from a pool of hyperimmune adults (PHIS) and a pool of malaria-naïve German donors were used as positive and negative controls, respectively. Samples were considered seropositive if they had an OD higher than the mean plus 3 SDs of the naïve controls.

### NK cell isolation

Venous blood was collected from healthy malaria-naïve donors, and PBMCs were subsequently isolated within 4 hours of collection using density gradient separation on a histopaque (1077 g/dl) monolayer. The PBMCs were washed twice in PBS, assessed for viability using trypan blue exclusion, and resuspended in cold RPMI 1640 medium supplemented with 10% fetal calf serum (Sigma-Aldrich) at a final concentration of 1.0 × 10^7^cells/ml. The NK cells were isolated from the PBMCs by depleting non–NK cell lymphocytes (negative selection) using an NK isolation kit as per the manufacturer’s instructions (Miltenyi Biotec). Briefly, 20 μl of a cocktail of mAbs were added to 1.0 × 10^7^ PBMCs and incubated for 30 min, followed by 30 min of incubation with 40 μl of microbeads at 4°C. The PBMC mixture was then passed through a MACs column followed by 5 ml of cold RPMI 1640 before collecting NK cells in the flow-through. The enriched NK cell mixture was washed, resuspended in fresh NK cell medium (RPMI 1640, 10% fetal calf serum, 1% penicillin/streptomycin, and 2 mM L-glutamine), and used on the same day. The NK cell isolation efficiency and cell purity (>90%) were confirmed by flow cytometry (fig. S1B).

### NK cell degranulation and intracellular cytokine assay

Frozen merozoites were thawed for 1 min in a water bath at 37°C. Merozoites (4.5 × 10^6^ merozoites per well) or recombinant *P. falciparum* antigens (30 ng/ml) were coated overnight in 96-well culture plates at 4°C. The wells were washed with PBS and blocked for 2 hours at 37°C with 1% casein in PBS. The coated plates were opsonized with heat-inactivated, prediluted (1:20) plasma samples for 5 hours at 37°C. Freshly isolated NK cells were added into each well (2.0 × 10^3^ NK cell per well). Anti-human CD107a PE (1:50), brefeldin A, and monensin (5 μg/ml) were added, and the plate was incubated for 18 hours at 37°C in 5% CO_2_. The NK cells were washed, centrifuged at 1500 rpm for 5 min, and resuspended in fluorescence-activated cell sorting (FACS) buffer (PBS, 1% bovine serum albumin, 1 mM EDTA, and 0.1% sodium azide). Their viability was assessed by staining with a fixable viability dye, eFluor 520, for 10 min at 4°C. The temperature was maintained at 4°C for all subsequent steps. A cocktail of anti-human mAbs comprising anti-CD56 allophycocyanin (APC), anti-CD3 phycoerythrin (PE)–Cy5, and anti-CD16 APC-Cy7 was used to stain the corresponding NK cell surface markers for 30 min. The cells were subsequently washed twice with FACS buffer, fixed with Cell Fix (BD Biosciences) for 10 min, and permeabilized with a permeabilization buffer (BD Biosciences) for 10 min. The permeabilized NK cells were stained intracellularly with anti-human IFN-γ PE-Cy7 for 1 hour in the dark. Last, the NK cells were washed thrice in permeabilization buffer, resuspended in FACS buffer, and stored at 4°C awaiting acquisition. Control wells included (i) unopsonized merozoites, (ii) merozoites opsonized with malaria-naïve plasma, (iii) merozoites opsonized with PHIS, (iv) merozoites incubated with PBS only, and (v) NK cells incubated with phytohemagglutinin (PHA; 1 ng/ml) and ionomycin (1 μg/ml; nonspecific NK cell stimulants). The acquisition was performed on the BD Biosciences FACSCalibur II high-throughput system in a 96-well plate format using FACSDiva. Data analysis was performed using FlowJo v10.

### Modified IIA-ADCC

About 20,000 NK cells per well, uninfected erythrocytes, and antibodies from malaria-naïve or malaria-exposed adults were resuspended in *P. falciparum* culture medium at a final hematocrit of 0.5% per well in a 96-well plate format. Synchronized schizonts (500-μl pellet) from *P. falciparum* strain NF54 was resuspended in 2500 μl of *P. falciparum* culture medium and filtered through a 1.2-μm membrane to release viable merozoites (64) and immediately added into each well (40 μl per well). The culture plate was incubated in a shaking incubator (50 rpm) for 30 min at 37°C to promote invasion. Purified polyclonal anti–rAMA-1 (1.5 ng per well), pooled malaria-naïve immunoglobulin (1.5 ng per well), and anti-MIG (1.5 ng per well) were tested. After 30 min of coincubation of merozoites, antibodies, and NK cells, unbound antibodies and free merozoites were removed by washing the culture twice in fresh *P. falciparum* culture medium. Subsequently, NK cells were removed using density gradient separation by gently adding histopaque (80 μl per well; 1077 g/dl) and spinning at 1800 rpm for 15 min without breaks. The culture was then washed twice, resuspended in *P. falciparum* culture medium (100 μl per well), and maintained for an additional 30 hours at 37°C. At the end of incubation, parasites were stained using the SYBR dye and enumerated by flow cytometry. Invasion inhibition was calculated against the proportion of the parasitemia recorded in the reference wells (test well without any antibody or NK cells).

### Flow data gating strategy and analysis

Flow data were analyzed using FlowJo v10.1 (TreeStar). The acquisition of stimulated NK cells was visualized in pseudo plots, with a time and single-cell gate used to exclude cell debris and double events, respectively. Lymphocytes were defined on the basis of size and granularity before gating on live cells defined as FITC^−^. Next, NK cells were identified as CD56^+^ and CD3^−^. Cells undergoing degranulation were identified as CD56^+^ and CD107a^+^ cells, whereas IFN-γ–secreting cells were defined as CD56^+^ and IFN-γ^+^ cells (fig. S7). The resulting gated cells were reported as proportions of the parent subset that were tabulated and exported for further analysis.

### Statistical analysis

Data were analyzed using Prism 8.07 (GraphPad) or Stata (version 14). The Mann-Whitney *U* test was used to compare medians between distinct pairs. The Kruskal-Wallis test was used to compare more than two groups and was supplemented with Dunn’s test for multiple comparisons. A nonparametric Spearman’s correlation was used to estimate the strength of pairwise correlations. The threshold level (analytical cutoff) above which Ab-NK was associated with protection was derived using maximally selected rank statistics (28). Briefly, the model evaluates the full range of possible cutoffs for the immunological readout (in this case, Ab-NK responses). It then selects the cutoff that maximizes the goodness of fit (log-likelihood) for the outcome variable (31). The responses were grouped into two groups (high and low). CHMI adults were treated when they had a parasite density of >500 Pf /μl or exhibited clinical symptoms and had any parasitemia. Junju children were considered to have experienced a malaria episode if they experienced a temperature of >37.5°C plus any parasitemia by blood smear (for children under 1 year) or a temperature of >37.5°C plus a parasite density of >2500 Pf/μl determined from a thick blood smear (for children older than 1 year). Both outcomes of interest for the two studies were fitted as binary variables in their corresponding models. Associations with clinical protection were assessed in both studies by comparing the incidence risk ratios between high and low Ab-NK responses using the modified Poisson regression model, which compares the relative risk between our two groups of interest during a specific time point of the follow-up period and Cox regression models over the 21 days of follow-up for the CHMI study and 6 months of follow-up for the child cohort (6, 32, 33). Potential confounders were adjusted to the respective models and included detectable levels of lumefantrine in the sample collected 1 day before challenge and the location of residence in the CHMI study. For the Junju cohort, we adjusted for age and schizont reactivity as a proxy for previous exposure.

## Supplementary Material

Data file

Figs S1-S7

MDAR

## Figures and Tables

**Table 1:** Ab-NK predicts protection after malaria challenge. The table shows the incidence risk and hazard ratios (IRR and HR) with 95% confidence intervals (95% CIs) of requiring treatment among challenged individuals who had high versus low Ab-NK activity responders estimated in the modified Poisson (first) and Cox regression (second) models, respectively. The “**#**” labeled panels show the mean with 95% CI of days when individuals who had high versus low Ab-NK activity responders required treatment as per the study protocol.

	Proportion of CHMI adults requiring treatment who had:			Univariate analysis			Multivariate analysis	
All CHMI adults (142)	High Ab-NK responses	Low Ab-NK responses		Ratio (95% CI)	*P* value		Ratio (95% CI)	*P* value
Incidence risk ratio (**cutoff value**)								
CD107a (**8.54**)	24% (24/97)	71% (32/45)		0.34 (0.23–0.51)	<0.000		0.41 (0.27–0.62)	<0.000
IFN-γ (**0.42**)	32% (41/125)	88% (15/17)		0.37 (0.27–0.50)	<0.000		0.49 (0.32–0.75)	0.0010
Time to treatment								
Hazard ratio								
CD107a	24% (24/97)	71% (32/45)		0.22 (0.13–0.38)	<0.000		0.25 (0.14–0.44)	<0.000
IFN-γ	32% (41/125)	88% (15/17)		0.14 (0.80–0.27)	<0.000		0.22 (0.11–0.45)	<0.000
Time to treatment (in days)#	Mean (95% CI)	Mean (95% CI)		*P* value				
CD107a	20 (20–21)	17 (16–18)		<0.000				
IFN-γ	20 (19–20)	15 (13–16)		<0.000				
Treated CHMI adults (*n* = 56)	CHMI adults who required treatment (*n* = 56)*							
	Top cluster (*n* = 40)	Bottom cluster (*n* = 16)						
Time to treatment (in days)#	Mean (95% CI)	Mean (95% CI)		*P* value				
CD107a	16 (14–18)	14 (13–15)		<0.000				
IFN-γ	16 (15–18)	13 (13–14)		<0.000				

*Ab-NK responses in treated CHMI adults were stratified into two clusters on the basis of Ab-NK cell responses (CD107a > 4.2; IFN-γ > 0.4) as the top (*n* = 40) or bottom (*n* = 16) clusters. All multivariate models were adjusted for low levels of antimalarial drugs (below minimal inhibitory concentrations) and the volunteers’ location of residence.

**Table 2:** Junju cohort baseline characteristics.

Age category (*n*)	<5 years (151)	>5 years (142)	0–12 years (293)
Sex *n* (% positive)			
Male	**71 (47%)**	**72 (49%)**	**143 (48%)**
Malaria slide–positive *n* (%)			
At cross-sectional sampling	**28 (23%)**	**52 (36%)**	**80 (27%)**
Malaria episodes *n* (% positive)	**77 (51%)**	**45 (31%)**	**122 (41%)**
Ab-NK activity seropositive *n* (%)			
CD107a^+^	**99 (65%)**	**101 (71%)**	**200 (67%)**
IFN-γ^+^	**8 (6%)**	**12 (10%)**	**12 (10%)**

**Table 3:** Ab-NK is associated with a reduced risk of clinical malaria episodes in children from Junju, Kenya. The table shows the incidence risk ratios (IRRs), hazard ratios (HRs), and 95% confidence intervals (95% CI) comparing the outcomes of children with high versus low Ab-NK responses in modified Poisson and Cox regression models, respectively. Data are presented as grouped analysis based on outcomes of interest: children who experience at least one clinical episode of malaria (i.e., fever over 37.5°C plus parasitemia above a predefined threshold, *n* = 122). The “**#**” labeled panel shows the mean and 95% CI for the time to malaria episode analysis comparing children with high versus low Ab-NK responders who had their first malaria episode during 6 months of follow-up. All multivariate models were adjusted for the potential confounding effects of age and previous *P. falciparum* exposure.

Junju children *n* = 293)	Proportion of children with			Univariate analysis			Multivariate analysis	
	High Ab-NK responses	Low Ab-NK responses		Ratio (95% CI)	*P* value		Ratio (95% CI)	*P* value
Acquired malaria episodes (*n* = 122)								
Incidence risk ratio (**cutoff values**)								
CD107a (**6.32**)	17% (10/56)	47% (112–237)		0.37 (0.11–0.67)	0.001		0.40 (0.22–0.71)	0.002
IFN-γ (**0.93**)	29% (15/51)	44% (107/242)		0.66 (0.42–0.97)	0.04		0.61 (0.39–0.97)	0.038
Time to treatment								
Hazard ratio								
CD107a	17% (10/56)	47% (112–237)		0.32 (0.17–0.61)	0.001		0.27 (0.13–0.52)	<0.000
IFN-γ	29% (15/51)	44% (107/242)		0.62 (0.36–0.99)	0.045		0.54 (0.31–0.97)	0.040
Time to first malaria episode (in days)#	Mean (95% CI)	Mean (95% CI)		*P* value				
CD107a	178 (160–195)	137 (126–147)		0.001				
IFN-γ	160 (139–182)	141 (131–151)		0.045				

## Data Availability

The study protocol and outcomes are published (23). Data used to generate the figures are included in data S1. Additional original data supporting this study’s findings are available from the data governance committee at KWTRP upon reasonable request (dgc@kemri-wellcome.org).
