# Supplementary material for: Anti-merozoite antibodies induce natural killer cell effector function and are associated with immunity against malaria
Source: Sci Transl Med. Author manuscript; Available in PMC 2024 Oct 3. (PMC7616656; doi:10.1126/scitranslmed.abn5993)
Supplement: Figs S1-S7 [file EMS198790-supplement-Figs_S1_S7.pdf]

Materials Design Analysis Reporting (MDAR)
Checklist for Authors

The MDAR framework establishes a minimum set of requirements in transparent reporting applicable to studies in the life sciences (see Statement of Task: doi:10.31222/osf.io/9sm4x.). The MDAR checklist is a tool for authors, editors, and others seeking to adopt the MDAR framework for transparent reporting in manuscripts and other outputs. Please refer to the MDAR Elaboration Document for additional context for the MDAR framework.

For all that apply, please note where in the manuscript the required information is provided.

Materials:

Newly created materials	indicate where provided: page no/section/legend)	n/a
The manuscript includes a dedicated "materials availability statement" providing transparent disclosure about availability of newly created materials including details on how materials can be accessed and describing any restrictions on access.	All the materials used to generate data from this study are commercially available. Samples from the two cohorts and additional original data that support the findings of this study are available from the data governance committee at KWTRP upon reasonable request; dgc@kemri-wellcome.org	

Antibodies	indicate where provided: page no/section/legend)	n/a
For commercial reagents, provide supplier name, catalogue number and RRID, if available.	Antibodies used for flow cytometry were:APC Mouse anti-Human CD56, (clone B159, cat. 555518,BD Bioscience); PE Mouse Anti-Human CD107a, (clone H4A3, cat. 560948, BD Bioscience); PE-Cy7 Mouse Anti-Human IFN-y, (clone B27, cat. 557643, BD Bioscience); PE-Cy5 Mouse Anti-Human CD3,(clone UCHT1, cat. 560835,BD Bioscience) and; APC-Cy7 Mouse Anti-Human CD16, (clone, 3G8, cat. 557758, BD Bioscience). Additionally, BD CompBeads Anti-Mouse Ig & negative control beads (cat. 552843 BD Biosciences) was included for compensation. FITC Viability dye (cat. L23101, ThermoFisher) stained dead cells.	

DNA and RNA sequences	indicate where provided: page no/section/legend)	n/a
**Short novel DNA or RNA including primers, probes:** Sequences should be included or deposited in a public repository.		X

Cell materials	indicate where provided: page no/section/legend	n/a
**Cell lines:** Provide species information, strain. Provide accession number in repository **OR** supplier name, catalog number, clone number, **OR** RRID.		X
**Primary cultures:** Provide species, strain, sex of origin, genetic modification status.		

Experimental animals	indicate where provided: page no/section/legend)	n/a
**Laboratory animals or Model organisms:** Provide species, strain, sex, age, genetic modification status. Provide accession number in repository **OR** supplier name, catalog number, clone number, **OR** RRID.		X
**Animal observed in or captured from the field:** Provide species, sex, and age where possible.		X

Plants and microbes	indicate where provided: page no/section/legend)	n/a
**Plants:** provide species and strain, ecotype and cultivar where relevant, unique accession number if available, and source (including location for collected wild specimens).		X
**Microbes:** provide species and strain, unique accession number if available, and source.		X

Human research participants	indicate where provided: page no/section/legend) or	n/a

	**state if these demographics were not collected**	
If collected and within the bounds of privacy constraints report on age, sex and gender or ethnicity for all study participants.	CHMI study: Healthy volunteers were recruited for the controlled malaria challenge study. They were aged between 18 and 45, females and males were equally eligible. We excluded participants with sickle cell trait as this is known to confer protection against malaria. We excluded volunteers with major infections, including HIV, Hepatitis B, and C, or those with clinical malaria. Full details are published in Kapulu 2021, JCI Insight.<br>Junju Cohort: Children aged between 1 and 12 years living in Junju were recruited at the onset of the malaria transmission season in 2008 and followed up for one year. Full details of the study participants are provided in the methods section.<br>For the Junju adults, healthy volunteers were invited to participate in seroepidemiological studies of malaria immunity. Details provided in the methods section	

Design:

Study protocol	indicate where provided: page no/section/legend)	n/a
If study protocol has been pre-registered, provide DOI. For clinical trials, provide the trial registration number **OR** cite DOI.	Clinical trial NCT02739763<br><br>The study protocol has been published https://www.ncbi.nlm.nih.gov/pmc/articles/PMC6871356/pdf/wellcomeopenres-3-17028.pdf.	

Laboratory protocol	indicate where provided: page no/section/legend)	n/a
Provide DOI **OR** other citation details if detailed step-by-step protocols are available.		X

Experimental study design (statistics details)		
**For in vivo studies:** State whether and how the following have been done	**indicate where provided: page no/section/legend. If it could have been done but was not, write not done**	**n/a**
Sample size determination	The samples used were from a previous controlled human malaria infection study. See the methods section. The sample size was based on the trial's primary outcome: to determine correlations between antibody levels to well characterized blood stage malaria antigens and in vivo parasite growth rates. Although 161 volunteers completed the challenge study, 19 were excluded from further analysis because they were either found to have antimalarial drugs in plasma (n = 12) or parasite genotypes other than the NF54 strain utilized in the challenge (n=7).We analyzed samples from all remaining volunteers (n = 142). These details are published and referenced (Kapulu 2021, JCI Insight).<br><br>For The Junju cohort. The sample size calculation was based on a previous study that assessed antibody-mediated respiratory burst in 300 children. They assumed this would give a 92% power to detect a 20% reduction in the risk of acquiring a malaria episode during the next season. In this study, we tested 293 out of the 300 samples.	
Randomisation	The CHMI study was not randomized since all the adults received the challenge, and later we compared their in vivo parasite growth. Additionally, The Junju study was a cohort observational study with no interventions.	
Blinding	The investigators were blinded during experiments with the sample vials labeled with unique identifiers, which were merged with the clinical data at the end of the analysis	
Inclusion/exclusion criteria	Nineteen of the CHMI adults were excluded from the analyzed data due to high levels of anti-malaria drugs above the effective dose in their plasma (n=12), or we detected the presence of a different parasite other than the challenging strain (n=7).<br>See methods section	

Sample definition and in-laboratory replication	indicate where provided: page no/section/legend	n/a
State number of times the experiment was replicated in laboratory.	The number of times each experiment was replicated in the laboratory is mentioned under each experiment.	
Define whether data describe technical or biological replicates.	The data describes technical and biological replicates.	

Ethics	indicate where provided: page no/section/legend	n/a

**Studies involving human participants:** State details of authority granting ethics approval (IRB or equivalent committee(s), provide reference number for approval.	The CHMI study was conducted at the KEMRI Wellcome Trust Research Programme in Kilifi, Kenya with ethical approval from the KEMRI Scientific and Ethics Review Unit (KEMRI//SERU/CGMR-C/029/3190) and the University of Oxford Tropical Research Ethics Committee (OxTREC 2-16). All participants gave written informed consent. The study was registered on ClinicalTrials.gov (NCT02739763), conducted based on good clinical practice (GCP), and under the principles of the Declaration of Helsinki.<br>The Junju cohort was originally recruited in 2005 and has been followed up for clinical episodes of malaria (Bejong 2006) In this study, we used samples collected in 2008. Ethical approval for the Junju study was provided by the Kenyan National and Scientific Ethics Review Committee protocol number 3149	
**Studies involving experimental animals:** State details of authority granting ethics approval (IRB or equivalent committee(s), provide reference number for approval.		X
**Studies involving specimen and field samples:** State if relevant permits obtained, provide details of authority approving study; if none were required, explain why.	See above section on human participants	X

Dual Use Research of Concern (DURC)	indicate where provided: page no/section/legend	n/a
If study is subject to dual use research of concern regulations, state the authority granting approval and reference number for the regulatory approval.		X

Analysis:

Attrition	indicate where provided: page no/section/legend	n/a
Describe whether exclusion criteria were preestablished. Report if sample or data points were omitted from analysis. If yes report if this was due to attrition or intentional exclusion and provide justification.	Exclusion criteria were preestablished before the CHMI study. The study protocol and exclusion criteria are published (Kapulu 2018).<br>Data from 19 out of 161 challenged adults was excluded and omitted from the analysis.<br>7 of the 161 volunteers were found to have non-PfNF54 stain (strain used to challenge) based on MSP2 genotyping and were excluded from further analysis (n=7). An additional 12 volunteers were excluded from the analysis because when we retrospectively measured their plasma levels for lumefantrine 7 days after the challenge, their plasma anti-malarial drug levels were higher than the minimum inhibitory concentration (MIC).<br>We, therefore, excluded the samples of these individuals (n =12) from further analysis. Thus, 142 of the 161 challenged volunteers were considered for further analysis.<br>Amongst the remaining volunteers, a proportion (n = 64) had low lumefantrine levels, i.e. below the minimum inhibitory plasma concentrations. Data from these individuals (n=64) were included in the downstream analysis. To minimize any potential confounding when assessing the association with protection, low levels of lumefantrine (n=64) were included as a confounder in the multivariate regression analysis. For more details, see the methods section.	

Statistics	indicate where provided: page no/section/legend	n/a
Describe statistical tests used and justify choice of tests.	Data were analyzed using Prism 8.07 (GraphPad) or Stata (version 14). The Mann-Whitney U test was used to compare medians between distinct pairs. The Kruskal-Wallis test was used to compare more than two groups and supplemented by Dunn's test for multiple comparisons. A nonparametric Spearman's correlation was used to estimate the strength of pairwise correlations.<br>The threshold level (analytical cutoff) above which ab-NK was associated with protection was derived using maximally selected rank statistics (Hothorn 2008). The responses were grouped into two groups (high and low). Associations with protection were assessed in both studies using the modified Poisson and Cox regression models.<br>Potential confounders were adjusted to the respective models and included; detectable levels of lumefantrine in the sample collected one day before the challenge and the location of residence in the CHMI study. For the Junju cohort, we adjusted for age and schizont reactivity as a proxy for previous exposure. See methods section	

Data availability	indicate where provided: page no/section/legend	n/a
For newly created and reused datasets, the manuscript includes a data availability statement that provides details for access or notes restrictions on access.	The study protocol and outcomes are published (Kapulu 2018). Additional original data supporting this study's findings are available from the data governance committee at KWTRP upon reasonable request; dgc@kemri-wellcome.org.	

If newly created datasets are publicly available, provide accession number in repository **OR** DOI **OR** URL and licensing details where available.		X
If reused data is publicly available provide accession number in repository **OR** DOI **OR** URL, **OR** citation.		X

Code availability	indicate where provided: page no/section/legend	n/a
For all newly generated custom computer code/software/mathematical algorithm or re-used code essential for replicating the main findings of the study, the manuscript includes a data availability statement that provides details for access or notes restrictions.		X
If newly generated code is publicly available, provide accession number in repository, **OR** DOI **OR** URL and licensing details where available. State any restrictions on code availability or accessibility.		X
If reused code is publicly available provide accession number in repository **OR** DOI **OR** URL, **OR** citation.		X

Reporting

MDAR framework recommends adoption of discipline-specific guidelines, established and endorsed through community initiatives. Journals have their own policy about requiring specific guidelines and recommendations to complement MDAR.

Adherence to community standards	indicate where provided: page no/section/legend	n/a
State if relevant guidelines (e.g., ICMJE, MIBBI, ARRIVE) have been followed, and whether a checklist (e.g., CONSORT, PRISMA, ARRIVE) is provided with the manuscript.		X
