# Supplementary material for: Anti-merozoite antibodies induce natural killer cell effector function and are associated with immunity against malaria
Source: Sci Transl Med. Author manuscript; Available in PMC 2024 Oct 3. (PMC7616656; doi:10.1126/scitranslmed.abn5993)
Supplement: MDAR [file EMS198790-supplement-MDAR.pdf]

Science Translational Medicine
AAAS

Supplementary Materials for

Anti-merozoite antibodies induce natural killer cell effector function and are associated with immunity against malaria

Dennis O. Odera *et al.*

Corresponding author: Faith H. A. Osier, f.osier@imperial.ac.uk

Sci. Transl. Med. **15**, eabn5993 (2023)
DOI: 10.1126/scitranslmed.abn5993

The PDF file includes:

Figs. S1 to S7

Other Supplementary Material for this manuscript includes the following:

Data file S1
MDAR Reproducibility Checklist

Supplementary Materials

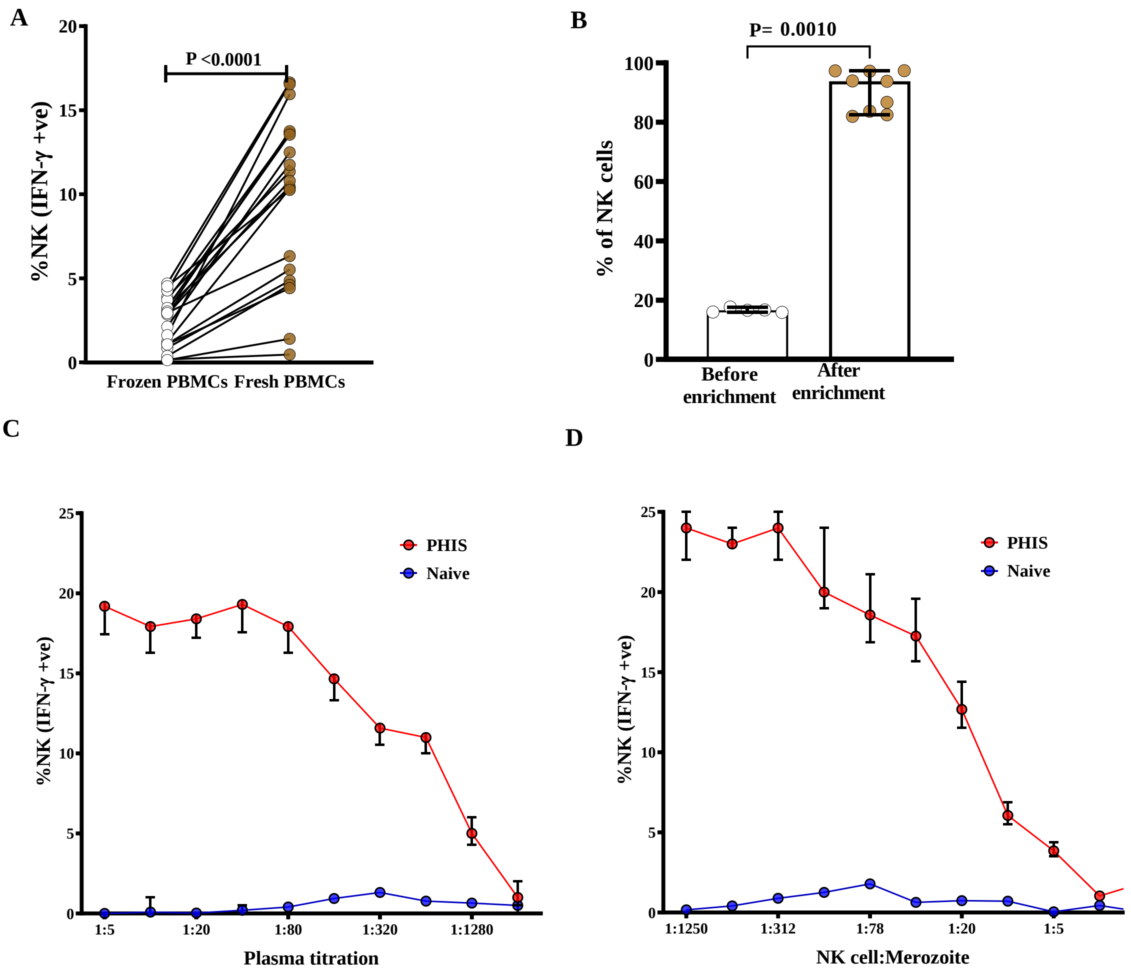

Supplementary Figure 1. Optimization of the ab-NK assay. (A) Higher IFN-γ production in NK cells isolated from fresh versus frozen PBMC. Plasma samples from Junju adults (N=25); P-value from the Wilcoxon matched-pairs signed rank test. (**B**) The proportion of NK cells (CD56+, CD3-) before (n=5) and after (n=9) NK cell enrichment. Error bars represent 95% CI of the median; Mann-Whitney test. Dose-dependent, NK cell IFN-γ production with increasing antibody concentration (**C**) and NK cell to merozoite ratio **(D)**. Merozoites were opsonized using a pool of hyperimmune serum (PHIS) from adults in Kilifi. Error bars represent a 95% confidence interval (CI) of the median of three replicates.

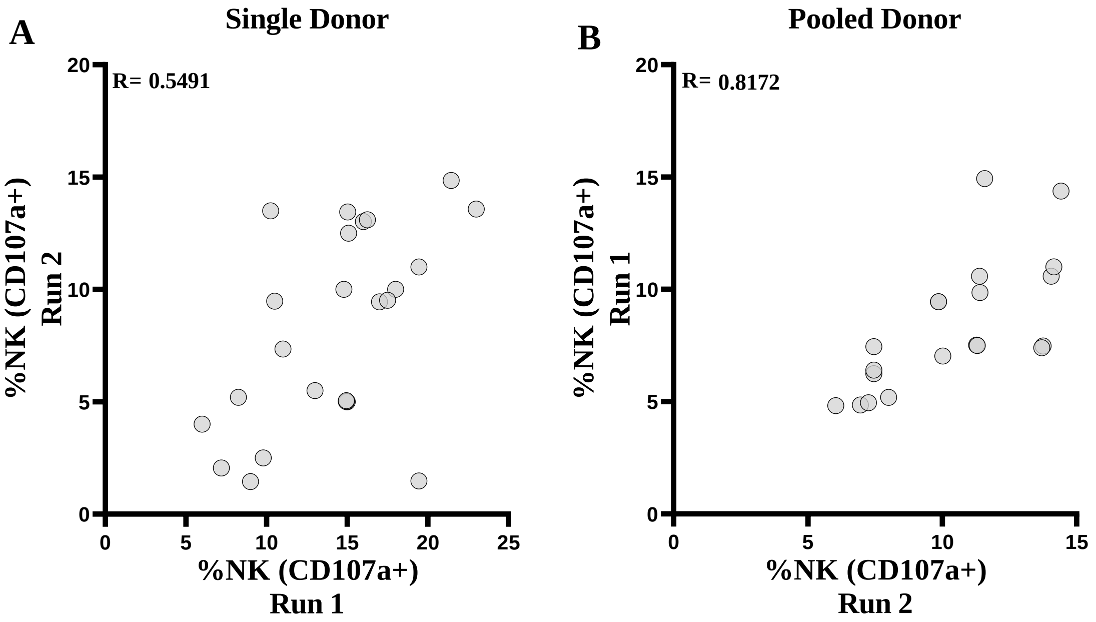

Supplementary Figure 2: **Pooling donor NK cells reduces inter-assay variation**: Pairwise correlation comparing the proportion of stimulated NK cell degranulating in two independent experiments conducted on different days using the same plasma samples from malaria-exposed adults (n=24). Left panel: the correlation between tests run using a single donor on day 1 (Run 1) versus responses against a second donor on day 2 (Run 2). Right panel; the assays were repeated using a pool of NK cells from 3 different donors each day (n=20). Spearman's R is indicated in the graph.

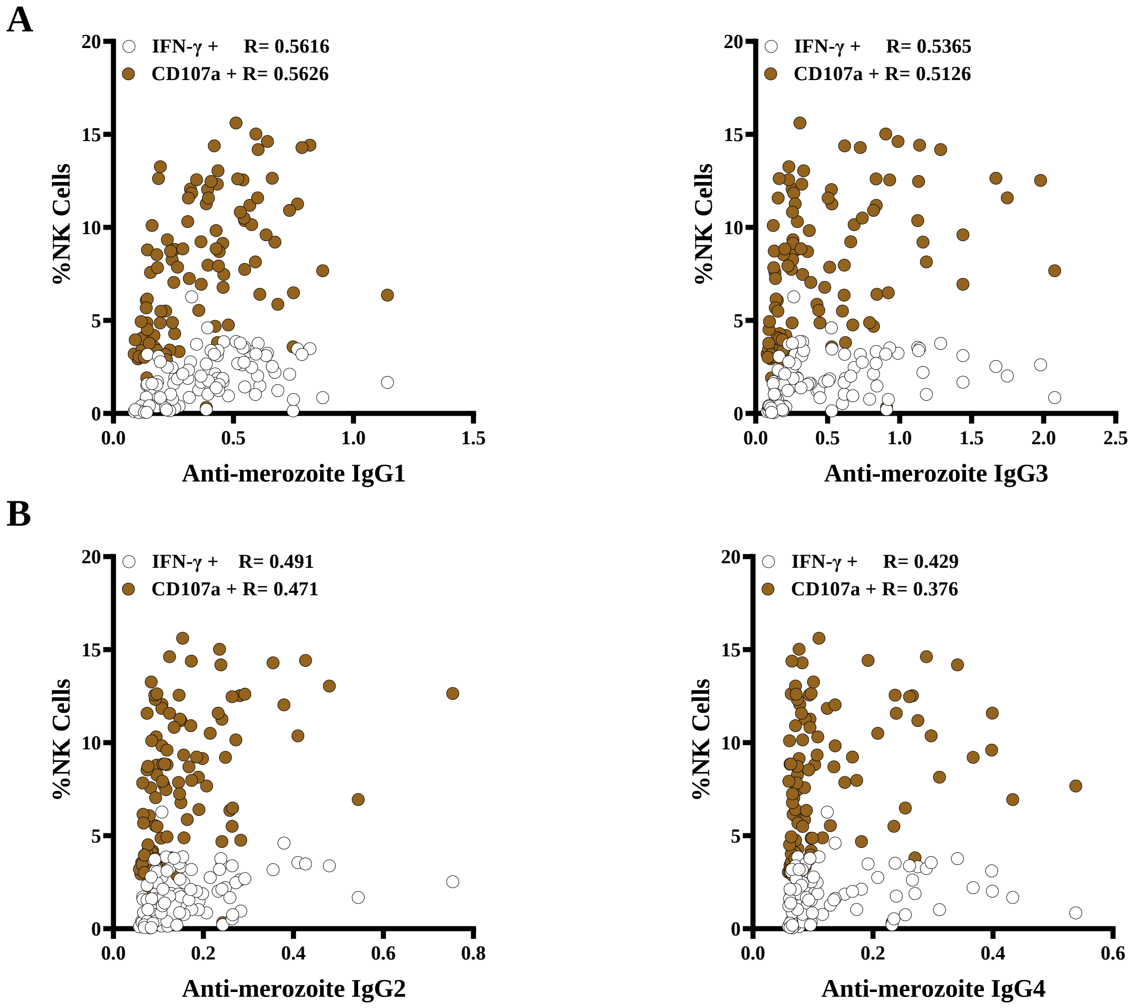

Supplementary Figure 3. Ab-NK activity correlates with anti-merozoite antibody binding Two-way scatter plots demonstrate the correlation between Ab-NK cell degranulation and IFN-γ production, respectively, versus IgG-binding antibodies against merozoites. Higher correlations were observed for cytophilic (A) compared to non-cytophilic (B) isotypes. The x-axis shows the ELISA optical density; Spearman's R, $P < 0.001$ for all comparisons; samples from the CHMI study (n= 142)

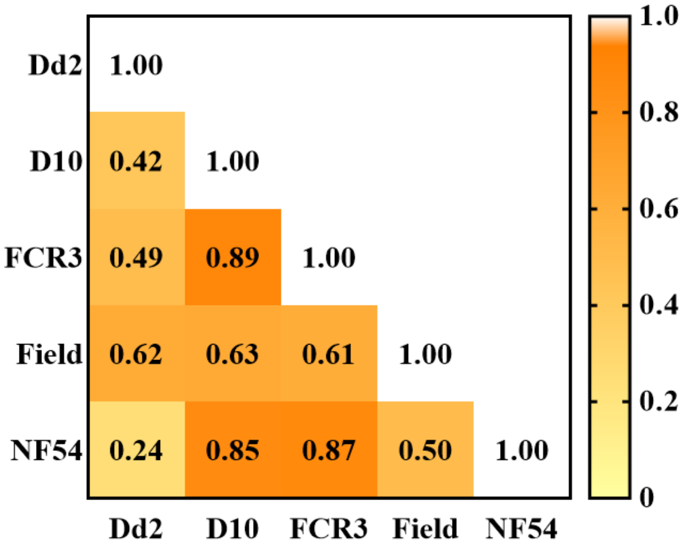

Supplementary Figure 4. Ab-NK activity mediates responses against multiple strains: Spearmans´ R correlation heatmap between the proportion of NK cells producing IFN-γ upon activation by opsonized merozoites from four *P. falciparum* strains of different geographical origin; West Africa (NF54), Gambia (FCR3), Papua New Guinea (D10) SE Asia (Dd2) and a recently adapted clinical isolate from Kenya (Field). Analysis was conducted in individual plasma samples from malaria-exposed adults from Junju county (n=20). Numbers in the heatmap indicate R values from Spearmans' correlation coefficient.

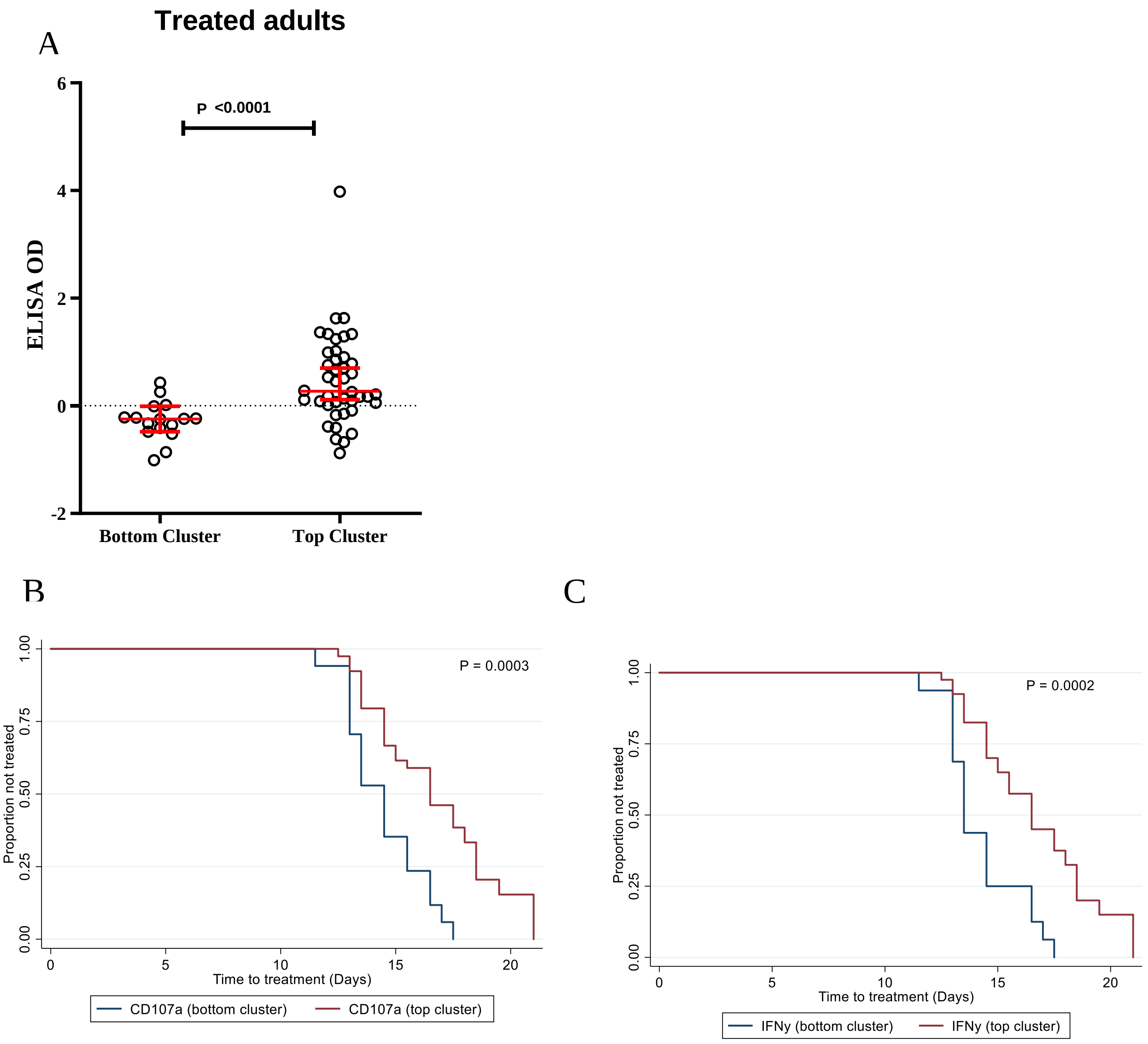

Supplementary Figure 5. Sub-analysis of ab-NK responses among the treated CHMI adults: CHMI adults treated during the follow-up (n=56) were categorized into two clusters based on their ab-NK responses. Individuals with low ab-NK responses (bottom cluster, <0,5% NK IFN-γ, n = 16) versus those with high responses (top cluster n=40). (A) Comparison of total IgG responses against *P. falciparum* merozoites between CHMI adults in the top versus bottom cluster. Mann Whitney t-test. Kaplan-Meier curves for the time to treatment for volunteers who received treatment grouped in the top cluster (red. n=40) versus bottom cluster (blue. n =16) for ab-NK degranulation (B) and IFN-γ production (C); log-rank test, P =0.0002 and P = 0.003, respectively.

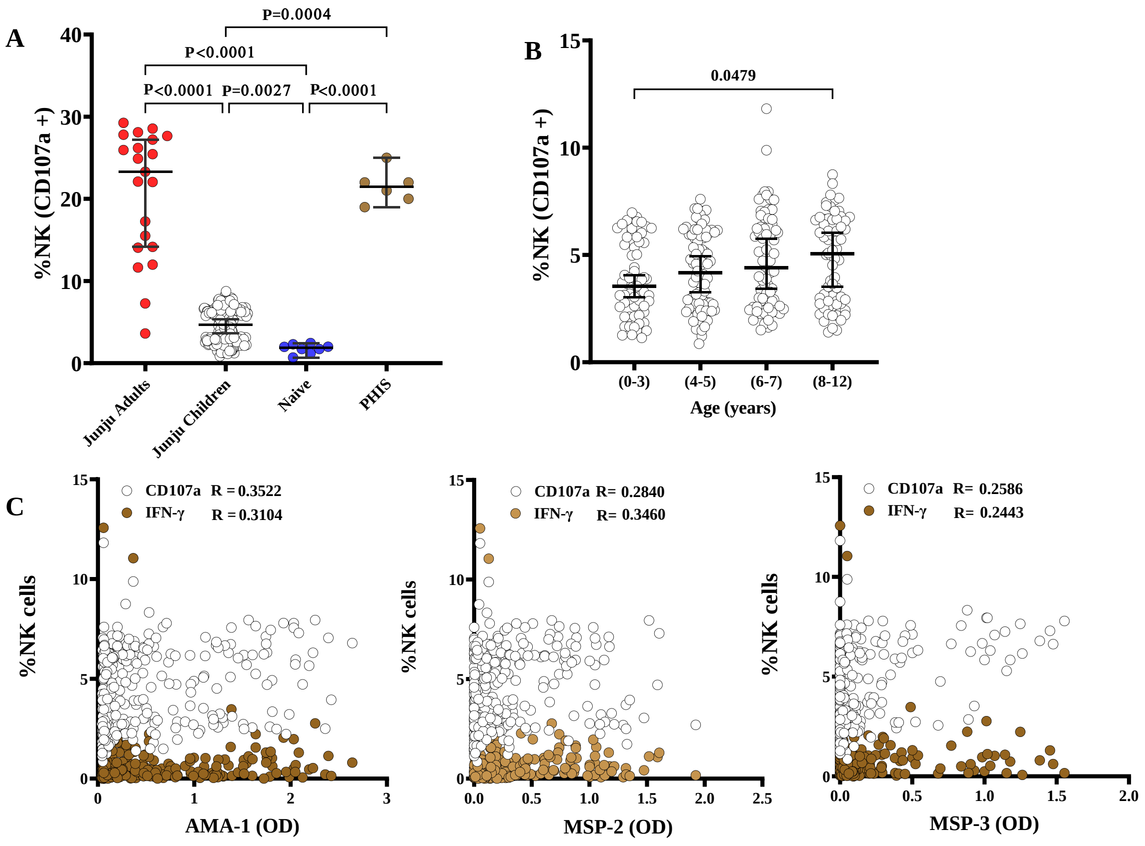

Supplementary Figure 6. Antibody-mediated NK cell activity in children: (**A**) Merozoite opsonizing antibodies from Junju adults (n=40) induced a significantly higher proportion of ab-NK cells degranulation compared to Junju children (n=293). Hyperimmune (PHIS) and malaria naive plasma were included as positive and negative controls, respectively. Error bars represent a 95% confidence interval of the median value; Kruskal-Wallis with Dunn's multiple comparison test. (**B**) Ab-NK cell degranulation in Junju children increased with age; Kruskal-Wallis with Dunn's multiple comparison test (n=293). (**C**) Spearman correlation between ab-NK cell degranulation or IFN-γ production and total IgG ELISA responses against recombinant *P falciparum* AMA-1, MSP2 or MSP3 antigens (n=293).

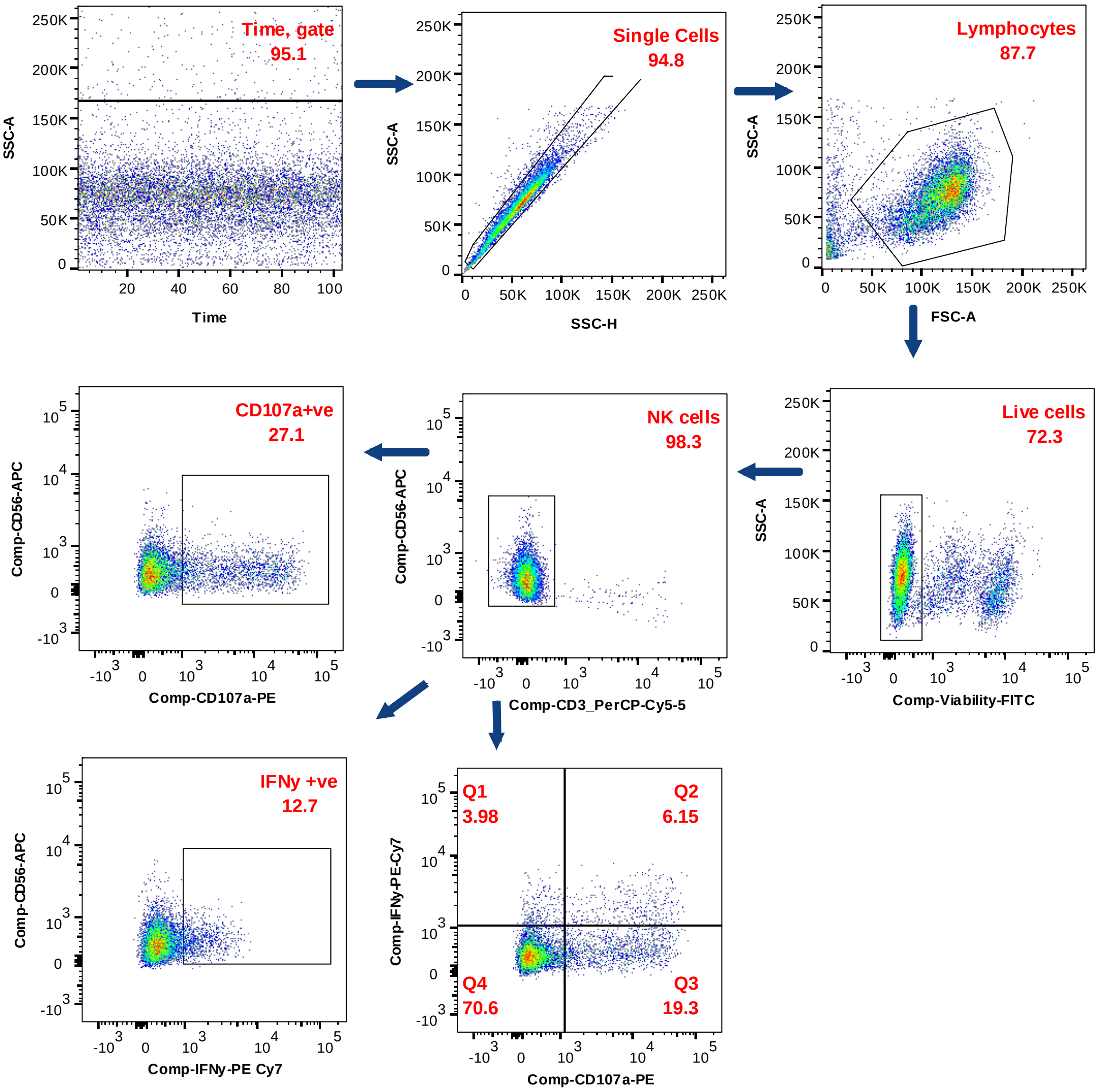

Supplementary figure 7. Flow cytometry gating strategy: The acquisition of stimulated NK cells was shown as pseudo plots with a time and single cells gate used to exclude cell debris and double events, respectively. Lymphocytes were defined based on size and granularity before gating on live cells defined as FITC-ve cells. Next, NK cells were identified as CD56+ve and CD3-ve cells. NK cells undergoing degranulation were identified as CD56+ve and CD107a+ve cells, while IFN-γ -secreting cells were defined as CD56+ve and IFN-γ +ve cells. Polyfunctional NK cells were defined as CD107a+ve and IFN-γ +ve cells. Side scatter area (SS-A), Side scatter height (SS-H), Forward scatter area (FS-A)
